# 

**Published:** 1861-11

**Authors:** 


AMERICAN.

The Principles and Practice of Obstetrics. By Gunning S. Bedford, M.D. New York, 1861. (From the author.)

The Morbid Effects of the Retention in the Blood of the Elements of the Urinary Secretion. By William Wallace Morland, M.D., etc. 8vo. Philadelphia, 1861.

Lectures on the Diseases of Women. By Charles West, M.D. Second American edition. 8vo. Philadelphia, 1861.

FOREIGN.

The Diseases of the Prostate, their Pathology and Treatment. By Henry Thompson, F.R.C.S. 8vo. London, 1861.

The Parasitic Affections of the Skin. By T. Mc Call Anderson, M.D. 8vo. London, 1861.

The Causes and Treatment of Imperfect Digestion. By Arthur Leared, M.D. 12mo., pp. 218. London, 1861.

The Sounds Caused by the Circulation of the Blood. By the same author. pp. 22. London, 1861.

Memoirs of Marshall Hall, M.D., F.R.S., etc. By his Widow. London, 1861. pp. 518.

On Fibrinous Deposition in the Heart. By Dr. B. Richardson. London, 1860. pp. 42.

A Treatise on the Surgical Diseases of the Eye. By H. Haynes Walton, F.R.C.S., etc. Second edition. London, 1861.

Advice to a Mother on the Management of her Offspring. By P. C. Chavasse, F.R.C.S. Sixth edition. London. pp. 267.

Climates for Invalids, etc. By A. Taylor, M.D. Third edition. pp. 283. London.

On the Influence of Light and Darkness on the Development, Growth, and Nutrition of Animals. By Horace Dobell, M.D. Reprint.

Bardeleben, Prof. A. Lehrbuch der Chirurgie und Operationslehre. 8vo. Berlin, 1861.

Scanzoni, Prof. F. W. Compendium der Geburtshülfe. 8vo. Wien, 1861.

Witmaack, Th. Lehrbuch der Arzneimittellehre. 8vo. Leipzig, 1861.

Bazin. Leçons sur la Scrofule considérée en elle-même et dans ses rapports avec la syphilis, etc. 2e édition. 8vo. Paris.

Bouillaud. De la congéstion cérébrale apoplectiforme dans ses rapports avec l'épilepsie. 8vo. Paris.

Lunel, A. B. Dictionnaire universel de médecine. T. 1. 1re partie. 12mo. Paris.

Coulon, le docteur A. Traité clinique et pratique des fractures chez les enfants; revu et précédé d'une préface par le docteur Marjolin. 8vo. Paris.

Duchenne (de Boulogne), le docteur G. B. De l'Électrisation localisée et de son application à la pathologie et à la thérapeutique. 2e édition, 8vo. Paris.

Fuster, J. Monographie clinique de l'affection catarrhale. 8vo. Paris.

Gallavardin, le docteur. Projet d'hôpitaux mixtes allopathiques et homœopathiques; projet de dispensaires mixtes; mémoire addressé à MM. les administrateurs des hôpitaux. 8vo. Paris.

Guardia, le docteur J. M. De l'étude de la folie. 8vo. Paris.

Hiffelsheim, le docteur. Des applications medicales de la pile de Volta, etc. 8vo. Paris.

Barrallier, le docteur A. M. Du typhus épidémique et histoire médicale des épidémies de typhus observées au bagne de Toulon en 1855 et en 1856. 8vo. Paris.

Bastin, le docteur A. De la congestion utérine pendant la grossesse. 4to. Paris.

Chassaignac, E. Traité clinique et pratique des opérations chirurgicales, ou Traité de thérapeutique chirurgicale. T. 1. 8vo. Paris.

Devay, Francis. De la médecine morale, précédée de réflexions sur la pratique de la médecine en général. 8vo. Paris.

Edwards, Milne. Leçons sur la Physiologie et l'anatomie comparée de l'homme et des animaux. Tome 6, 2e partie. Appareil Digestif. 8vo.

Giraud-Teulon, le docteur F. Physiologie et pathologie fonctionnelle de la vision binoculaire, suivies d'un aperçu sur l'appropriation de tous les instruments d'optique à la vision avec les deux yeux, l'opthalmoscopie et la stéréoscopie. 8vo. Paris.

Issartier, le docteur F. De l'alcoolisme moderne. 8vo. Paris.

Ladreit de Lacharrière, le docteur J. Des paralysies syphilitiques. Grand 8vo.

Métaxas, le docteur S. J. De l'exploration de la rétine et des altérations de cette membrane visibles à l'opthalmoscope. 4to., avec 3 planches.

Gourdin, A. H. C. Du traitement de la tuberculose. 4to.

### INDEX.

Abbot, S. L., occlusion of vagina, 702.
Abegg, Dr., galactorrhœa, 701.
Abortion, cold a principal cause of, 897.
in cows, caused by ustilago madis, 1121.
with retained placenta, treatment of, 894.
Abortions, contributions to history of, 341.
Abscess, abdominal, 878.
hepatic, communicating with lung, 366.
pelvic, 878.
Absorption, cutaneous, experiments on, 708.
Academy of Medicine of Paris, prizes of, 379.
Aceton, in affections of the larynx, 157.
Acton, W., post-mortem on Lord Chancellor Campbell, 1103.
Adams, F., death of, 575.
W., reparation in human tendons, review of, 444.
Aemilia rigidula, 713.
Agnew, D. H., operation for radical cure of inguinal hernia, 117.
Ailanthus glandulosa, 714.
Albuminoid substances, decomposition of, 162.
Albuminuria, diseases of kidneys accompanied by, 887.
Alcaptone in the urine, 912.
Alcohol and anæsthetics, 707.
its action and uses, 1121.
Aldridge, J. H., adhesion of the bowels to the uterus, 173.
Aletris farinosa, effects of, 110.
Alkalies, influence of on circulation, the liver, etc., 908.
Althaus, Dr., electricity as an emmenagogue, 903.
Amaurosis, lead, 313.
Amenorrhœa, treatment of, 901, 903.
American biography, 408.
American Medical Association, 571.
transactions of, review of, 603, 1005.
Ammonia, phosphate of, in rheumatism, 711.
Amputation, statistics of, at St. George's Hospital, 673.
Anacahuite wood, 539.
Anæsthesia, recurrent, from effusions within the spinal canal, 517.
Anæsthetics and alcohol, 707.
Anatomist, pocket, by Hilles, notice of, 64.
Anatomy, human, Leidy's elementary treatise on, review of, 52.
microscopic, Kölliker's, 831.
of the arteries, etc., 448.
Anchieta salutaria in skin diseases, 710.
Andrews, Dr., death of, 383.
Aneurism, at apex of heart, 552.
digital compression in, 114, 377.
gluteal, cases of, 879.
of the aorta, 169, 552, 553.
destroying spinal vertebræ, 552.
popliteal, cured by flexion, 683.
digital compression in, 508.
with rupture of the aorta, 363.
Aneurismal varix after pressure for the cure of aneurism, 881.
Aniline, operation of, upon the animal body, 910.
Animals for human food, composition of, 538.
Antimony, sulphide of, in cardiac affections, 156.
Anus, fissure of, treated by dilatation of the sphincter, 508.
preternatural, case of, 906.
Aorta, aneurism of, 169, 552, 553.
Aortic valves, disease of, with ascites, 720.
ossification of, 1103.
Appendix vermiformis, case of perforation of, 172.
foreign body in, 172.
Aran, Dr., death of, 575.
cancer of the uterus, 136.
Archiv für Klinische Chirurgie, notice of, 65.
Armor, S. G., appointment of, 759.
Army medical board of examiners, 571.
surgeons, examining board of, 184.
surgeons in the field, 765.
Arnott, C., rigidity of the os uteri, 522.
Arsenic, detection of, 913.
in indigestion, 352.
Arsenical poisoning, 717.

Arsenious acid in the metamorphosis of matter, 537.
Artemisia contra, physiological action of, 910.
Arum, poisoning by, 715.
Asphyxia, researches on the hot bath, etc. in, 884, 1105.
Asthma, pathology of, 1106.
Atkinson, W. B., abstracts by, 130, 333, 522, 697, 894, 1107.
Atmospheric pressure, influence of change of, 907.
Attfield, Mr., on sugar in the urine, 545.
Auditory nerve, lesions of, producing cerebral symptoms, 533.
Auerbach, L., muscular contractions from irritation, 152.

Backus, H., pathological phenomena generalized, notice of, 836.
Barnes, R., placenta prævia, 1111.
on premature labor, 1112.
Barry, M., case of ovarian dropsy, 898.
Balch, G. B., gunshot wound of the heart, 681.
Baly, W., death of, 575.
Barstow, J. W., statistics of insanity, 695.
Barwell, R., excision of the head of the femur, 512.
Bath, Turkish, in insane asylums, 764.
Baunscheidtismus, 942.
Bazin, M., use of phenic acid, 1117.
Beale, Dr., acute pericarditis with effusion, 332.
Beau, M., puerperal vaginitis, 705.
Becquerel, A., treatise on electricity, review of, 249.
Bedford, Prof., work of, in French, 571.
work on obstetrics, review of, 1035.
Begbie, J. W., pulmonary cancer, 718.
Belcher, T. W., cancer of the gall-bladder, 557.
Bell, B., perforation of the stomach, 689.
J., identity of typhus and typhoid fevers, 128.
John, on the importance, etc., of sanitary measures to cities, review of, 832.
Joseph, pulsating tumors in the orbit, 1084.
Bellevue Hospital College, 569, 759.
Bennett, G. J., cold a principal cause of abortion, 897.
Bernard, C., election of, 571.
on muscular poisons, 149.
D., euphorbia peplus, 716.
Bernardet, M., luxation of the radius, 1088.
Beronius, V. M., on diseases of the brain, 166.
Bidwell, E. C., new test for diabetes, 545.
Birth, live, what constitutes, 1123.
Blackman, G. C., handbook for the military surgeon, review of, 818.
popliteal aneurism cured by digital compression, 508.
Blenorrhagia, copaiva, etc. in, 1119.
ergot in, 352.
Blind persons in Ireland, 765.
Blood-letting in Italy, 957.
Bloody sweat, a case of, 515.
Boedecker, M., alcaptone in the urine, 912.
glucose with chondrine, 912.
Bone, regeneration of, 1087.
Book about doctors, 577.
Borailler, M., essence of valerian, 711.
Botalli, opera omnia, review of, 788.
Bouchardat, M., kousso in tape-worm, 350.
Bourrousse, Dr., iodine in tubercular meningitis, 1117.
Bouvier, M., luxations of the radius, 1088.
Bowditch, Dr., patency of foramen ovale, 551.
Bowels, adhesion of, to the uterus, 173.
Bozeman's button suture in lithotomy, 121.
Bradford, J. T., urinary calculus in a child, 669.
Brain, diseases of, 166.
organic disease of base of, 1098.
researches on the functions of, 534.
Braune, W., a case of preternatural anus, 906.
Breast, inflammation of, 896.
treatment of inflammatory affections of, 137.
Brigade surgeons, appointment of, 956.
Bright's disease with eclampsia, 133.
Briquet on hysteria, review of, 193.
Bristowe, Dr., modes of death in typhoid fever, 1094.
Brockendahl, M., reduction of inversio uteri, 1115.
Brodie, Sir B., operation on, 378.
Bronchitis as a cause of lung affections, 834.
Brougham, J. P., on sun-stroke at Lucknow, 124.
Brown-Séquard, M., Croonian lecture by, 762.
lectures on central nervous system, review of, 811.
lesions of the auditory nerve, 533.
symptoms of disease of base of brain, 1098.
Bruce, Dr., intra-uterine small-pox, 342.
Brunonianism, Toddism, etc., by Prof. Gross, 66.

Bryant, T., on compound fractures, 504.
Bryson, Dr., yellow fever in Jamaica, 690.
Buck, H. J., case of hermaphroditism, 558.
Bucknill, Dr., Psychology of Shakspeare noticed, 1126.
Budge, Prof., the cæliac and mesenteric plexus, 145.
Bull Run, battle of, a visit to the wounded, 949.
Bury, Dr., digital compression in hemorrhage, 116.
F., statistical account of 476 cases of acute rheumatism, 893.
Busch, Prof., on reduction of dislocations, 1089.
Butcher, R. G. H., extirpation of a scirrhous eyeball, 509.
Byford, W. H., inflammatory affections of the breast, 137.
ovariotomy, 369.

CADY, G. P., opium in puerperal peritonitis, 897.
Cæsarean section, case of, 524.
Calculus, cystic, in a dog, 311.
Campbell, Lord Chancellor, post-mortem on, 1103.
Camplin, J. M., on diabetes, notice of, 266.
Cancer, epithelial, of the larynx, 168.
of the lip, report on, 119.
of the tongue, report on, 119.
of the gall-bladder, 557.
of the liver, 365.
of the lungs and mediastinum, 718.
of the œsophagus, 365.
obscure cases of, 920.
of the uterus, 136.
Cangella, M., arum poisoning, 715.
Canton, Mr., aneurism of the heart, 552.
Carroll, T., causes of the venous circulation, 453.
Cartilages of joints, ulceration of, 175.
Cataract spontaneously cured, 317.
Caylus, Prof., solanum dulcamara, 709.
Cellular pathology of Virchow, review of, 385.
Cerate, substitute for, in dressing wounds, 1090.
Cerebellum as the centre of co-ordination, etc., 534.
Cervantes an M.D., 751.
Cervix uteri, removal of, 1114.
Chambers, T. K., a case of bloody sweat, 515.
Channing, W., cystic tumors of the vagina, 1114.
Chauveau, A., application of the hemodrometer, 531.
Cheever, D. W., value and fallacy of statistics in disease, 892.
Chemistry, physiological, Day's, notice of, 1036.
Children, surgical diseases of, 438.
Chipley, W. S., a warning to fathers, etc., notice of, 636.
Chlorate of potassa in phthisis, 351.
Chloroform, ether, etc., new way of giving internally, 1120.
Chlorosis, especially in children, 530.
Cholera in Calcutta, 764.
Chondrine with glucose, formation of, 912.
Christian, Dr., restoration of the apparently drowned, 519.
Chuguiragua in intermittents, 710.
Churchill, F., midwifery, review of, 46.
Ciliary muscle, paralysis of, 688.
Cinchonine in intermittents, 1117.
Circulation in the horse, rapidity of, measured, 531.
Civiale, Dr., clinical experience on stone, 506.
Clark, A., ulceration, etc., of the colon, 367.
H. G., draft of a sanitary code for cities, review of, 832.
Clay, C, placenta prævia, 1111.
J., detachment of the placenta, 335.
on diseases of the ovaries, review of, 417.
Clifton Hall, visit of physicians to, 956.
Climate of Egypt, 959.
Coagulation, Zimmerman's views on, 538.
Coal-tar emulsion, 712.
Cobb, J., death of, 186.
Coca, observations on, 156.
Cod-liver oil, taste and smell of, 350.
Cæliac and mesenteric plexus, functions of, 145.
Cold, a principal cause of abortion, 897
death by, morbid appearances in, 173.
Collodion in phlegmasia dolens, 897.
Colon, ulceration and stricture of, 367.
Combe, A., on infancy, review of, 33.
Conceptions in anæmia, frequency of, 702.
Conservative medicine, by Flint, 1038.
Constantinople, Imperial Medical Society of, prizes of, 572.
Consumption, anacahuite wood in, 541.
in the Hebrides, 125.
Cooke, M. C., kukui oil, 542.
Cooper, G. F., statistics of compound fracture, 505.
statistics of amputations, 673.

Cooper, G. F., statistics of operations for strangulated hernia, 872.
Coote, C., death of, 191.
H., on the radical cure of hernia, 877.
exostosis of a vertebra, 680.
Copaiva and matico, 1118.
distilled water of, 1119.
Corpora amylacea, theory of, 727.
Corpus callosum, hemorrhage into, 919.
Corrigan, D. J., aortitis with peritonitis, 720.
Corse, J. M., fungus hæmatodes of the uterus, etc., 369.
Cortese, Dr., gunshot wounds, 681.
Cosmetics, poisonous, 958.
Cotyledon umbilicus in epilepsy, 154.
Coulon, M., sero-sanguineous cyst of the nates, 559.
Coxalgia, resection in, 682.
Creatin and creatinine in health and disease, 345.
Creosote vaginal injections, 135.
Crede, Dr., removal of the placenta by uterine action, 895.
Cuckoo-pint, poisoning by, 715.
Culinary campaign, Soyer's, reviewed, 1023.
Currents and counter-currents, by Holmes, review of, 235.
Cusack, Mr., death of, 1137.
Cutter, Mr., encephaloid tumors of the neck, 550.
Cyanide of potassium, poisoning by, 359.
Cystine calculus in a dog, case of, 311.
Cysts, serous, of the breast, 923.
Czermak on the laryngoscope, review of, 262.

Da Costa, J., tubercular disease of the heart, 362.
D'Almeida, A. B., case of popliteal aneurism, 363.
Dalton, J. C., Jr., human physiology, notice of, 449.
functions of the cerebellum, 534.
David, A. H., small-pox in mother and child, 706
Davies, R., five cases of neurotomy, 121.
Day, G. E., physiological chemistry, notice of, 1036.
Deafness, a peculiar form of, 532.
Deaths of medical men, (see Necrological notices.)
causes of, in England, in 1660 and 1860, 1100.
Debout, M., electuarium for blenorrhagia, 1119.
Debrou, M., on ectropion and its treatment, 316.
Dechambre, M., connective tissue of nerves, 726.
Delirium tremens, digitalis in, 157.
Demeaux, M., coal-tar emulsion, 712.
Dental colleges, students in, 569.
hospital, dinner of, 763.
national, in England, 958.
Désormeaux, M., a substitute for cerate, 1090.
Desprès, M., death of, 191.
Devillers, M., shortness of the umbilical cord, 142.
Diabetes and its treatment, 266.
test for, 545.
Diaphragm, congenital deficiency of, 364.
influence of the pneumogastric and superior laryngeal nerves on, 908.
Dickinson, W. H., empyema, etc., in the mediastinum, 169.
Digital compression in aneurism, 114, 377.
in hemorrhage from the brachial artery, 116.
in popliteal aneurism, 508.
Digitalis in delirium tremens, 157.
in organic affections of the heart, 909.
Diphtheria, fatality of, 185.
its nature and treatment, 330.
with puerperal fever, 700.
Diseases of the kidneys accompanied by albuminuria, 887.
Dislocation of the knee-joint, intra-uterine, 116.
Dislocations, reductions of, 1089.
Douche, uterine, in galactorrhœa, 701.
Dromgoole, J. P., effects of aletris farinosa, 110.
Drowned, restoration of animation in, 519, 1105.
Druitt, R., puerperal fever with diphtheria, 700.
Drysdale, T. M., rupture of ductus communis choledochus, 723.
Duchenne, M., locomotory ataxy, 694.
Dumontpellier, M., emboli of the pulmonary artery, 721.
Duncan, M., warts on the vocal cords, 717.
Dunglison, Richard J., abstracts by, 124, 322, 513, 686, 884, 1092.
reflections on exanthematic typhus in Liverpool, 861.
Dupierris, E., on strictures of the urethra, notice of, 63.
Durr, M., alcaptone in the urine, 912.

Eade, P., aneurism of the aorta, 553.
Ear, caries of petrous bone in diseases of, 685.

Eclampsia of pregnancy, etc., with Bright's disease, 133.
Écraseur in lithotomy, 764.
Ectropion treated by closure of the lids, 316.
Edmunds, J., case of Cæsarean section, 524.
Egypt, climate of, 959.
Egyptian fever in Liverpool, 690.
Eisenmann, Dr., local action of savin, 911.
Electricity as an emmenagogue, 903.
as a therapeutical agent, 249.
Elliot, G. T., Jr., rupture of uterus, 523.
Elmer, W., visiting list, notice of, 64.
Emboli of the pulmonary artery, 721.
Empyema, case of, 363.
with malignant disease, 169.
Encephaloid tumors of the neck, 550.
Endocardial murmurs, effect of posture on, 686.
Epidemic tracked, 690.
Epidemics and endemics, report on, by Dr. Gibbs, 83, 469.
Epidemiological Society's transactions, 452.
Epilepsy, analysis of fifty-two cases of, 514.
cotyledon umbilicus in, 154.
woorara in, 352.
Epileptiform convulsions from bleeding, 520.
Episcopal Hospital, notice of, 570.
Epithelial cancer. (See cancer, epithelial.)
Ergot, action of on the fœtus, 1109.
in blenorrhagia, 352.
Erichsen, J. E., science, etc., of surgery, notice of, 451.
Erlenmeyer, M., on albuminoid substances, 162.
Erysipelas, infantile, 904.
Escolar, M. S., on chuguiragua, 710.
Ether and chloroform compared, 679.
patent, 183.
Euphorbia lathyris as a poison, 716.
peplus as a poison, 716.
Eve, Dr., position of, 759.
Exanthematic typhus in Liverpool, reflections on, 861.
Excision of joints, results of, 938.
of the femur, 512.
Exostosis of the seventh vertebra, 680.

FALCK, PROF., on santonin, 160.
Fauconnet, M., antimony in heart affections, 156.
Favrot, Dr., matico with copaiva, 1118.
Favus, observations on, 328.
Female doctors, American, 765.
nurses, 758.
Fergus, Dr., iron and quinine, 712.
Fergusson, Mr., lithotomy in children, 1091.
Ferri carbonas effervescens, 1120.
Fever, treatise on, by Lyons, 600.
typhoid and typhus. (See typhoid and typhus.)
Finley, Dr., appointment of, 759.
Finnell, Dr., case of aneurism of the aorta, 169.
Fischer, E., abstracts by, 154, 349, 539, 709, 909, 1117.
Fischer, M., glucose with chondrine, 912.
Fish poisons, remarks on, 165.
Flint, A., analysis of one hundred and thirty-three cases of pneumonia, 322.
conservative medicine, 1038.
Florida, medical topography of, 646.
Foramen ovale, patency of, 329, 551.
Forceps, timely use of, 1107.
fœtal head as an obstruction to, 1109.
Forster, J. C., surgical diseases of children, review of, 438.
Foster, M., a new remedy for hooping-cough, 906.
Foucher, M., strychnia in prolapsus ani, 707.
Fountain, E. J., chlorate of potassa in phthisis, 351.
death of, 575.
placenta prævia, 523.
Fox, W. T., phlegmasia dolens, 339.
Fracture, compound, contributions to the subject of, 504.
statistics of, 505.
of patella, Malgaigne's hooks in, 683.
Fractures of the lower extremity, anterior suspensory apparatus for, 882.
France, statistics of insanity in, 927.
Francis, J. W., death of, 380.
Franque, Dr. O. Von, puerperal epidemic, 134.
Frerichs, syphilis in the liver, 722.
Frost, effect of on public health, 379.
Fungi, poisoning by, 359.

GALACTORRHŒA treated by uterine douche, 701.
Gall-bladder, congenital absence of, 925.
Garner, R., on excision of joints, 938.
Garratt, A. C., electro-physiology, etc., review of, 250.
Gay, Dr., case of uterine hæmatocele, 900.
Germain, M., arsenic in indigestion, 352.
Gibb, G. D., saccharine fermentation of the milk, 1116.

Gibbs, Dr., rank of, 759.
O. C., report on epidemics and endemics, 83, 469.
Gilbert on animal food, 538.
Gill, J. B., epitome of surgery, review of, 60.
Glasgow, medical profession in, 1135.
Gleet, local remedies in, 666.
Globularia alypum as a purgative, 349.
Glucose with chondrine, formation of, 912.
Glycerin as a disinfectant, 714.
and camphor in suppression of milk, 337.
Goldschmidt, Dr., fungosities of the uterus, 900.
Goodell, W., Turkey medically considered, 486.
Greenhalgh, R., removal of the cervix uteri, 1114.
Griscom, J. M., report upon sewerage, water supply, etc., review of, 832.
Gross, Prof. S. D., American medical biography, 66.
review of, 408.
Brunonianism, Toddism, and other isms, 66.
cases in surgery, 656.
editorial valedictory by, 1125.
manual of military surgery, notice of, 642.
proposed work on scalp, etc., 185.
sketch of life of Paré, 1059.
Gross, S. W., abstracts by, 114, 313, 504.
cystine calculus in a dog, 311.
Gunshot wound of the cranium, trepanning in, 679.
of the heart, 681.
Gunshot wounds in loading, 681.
resection in, 682.

HÆMATINE as a test, 715.
Hæmatocele, uterine, case of, 899, 900.
Haldane, Dr., hemorrhage into the corpus callosum, 919.
Haller, C., on coca, 156.
Hamilton, F. H., a treatise on military surgery, review of, 818.
Hammond, W. A., nitric acid in intermittents, 541.
on a new species of upas, 537.
on uræmic intoxication, 536.
Hamolecki, Dr., phosphate of ammonia in rheumatism, 711.
Hanbury, D., anacahuite wood as a remedy, 539.
Harley, Dr., on sugar in the liver, 147.
Harris, C., death of, 190.
T., death of, 573.
W., death of, 573.
Harriss, J., glycerin and camphor in suppression of milk, 337.
Hartshorne, H., memoranda medica, notice of, 62.
Hasselt, V., on santonin, 160.
Hayne, A. P., occlusion of vagina, 701.
Head, injury of, with loss of brain, 507.
Heart affections, antimony in, 156.
anomaly of the, 719.
digitalis in, 909.
hypertrophy of, 1103.
penetrating wound of, 940.
tubercular disease of walls of, 362.
Heat-cure, 353.
Heath, C., cysts simulating femoral hernia, 1090.
Hemodrometer applied to the horse, 531.
Hemorrhage, digital compression in, 116.
from a large artery, treated by digital compression, 116.
uterine, persulphate of iron in, 132.
Hereditary disease, sexual limitation in, 1095.
Hermaphroditism, case of, 558.
Hernia, femoral, cysts simulating, 1090.
inguinal, radical cure of, 117, 877.
strangulated, statistics of operations for, 872.
Hétet, Dr., ailanthus glandulosa, 714.
Hewitt, G., fœtal head as obstruction to forceps, 1109.
Hewson, A., appointment of, 378.
Hicks, J. B., excrescence of os uteri removed, 527.
Hill, A., small-pox in the mother and infant, 705.
Hilles, M. W., pocket anatomist, notice of, 64.
Hindu system of medicine, 616.
Hip-joint, affections of, from uterine disease, 141.
Histology, human, Morel's, 829.
Hodge, Prof., on diseases of women, review of, 220.
H. Lenox, serous cysts of the breast, 923.
Holmes, O. W., currents and countercurrents, review of, 235.
T., statistics of amputations, 673.
statistics of operations for strangulated hernia, 872.
Hong Kong, diseases of, 1104.
Hooping-cough, treatment of, 905, 906.
Hoppe, Dr., hip-joint affections from uterine disease, 141.
Horner, G. R. B., medical topography of Florida, 646.
Hospital practice, handbook of, 640.
Hot-bath, employment of, in cases of asphyxia, 884, 1105.

Howe, F. A., foreign body in the appendix cæci, 172.
Humerus, luxation of head of, reduced by manipulation, 510.
Hunter, C., hot bath in asphyxia, 1105.
 J., irritation of the urethra in females, 903.
 ovarian disease with pregnancy, 700.
Hutchinson, J., on ring-worm, 328.
 recovery from tetanus, 675.
 report on epithelial cancer of the lip and tongue, 119.
 J. C., enlarged ovary and ruptured uterus, 899.
Hydrogen, properties of peroxide of, 143.
Hygiene, therapeutical, review of Ribes's work on, 961.
Hypochondriac delirium preceding paralysis, 517.
Hyrtl, Prof., sebaceous tumors simulating piles, 316.
Hysteria, review of Briquet on, 193.
 veratrum viride in, 903.

ICTERUS with fatty degeneration, 556.
Idiots, report of Penna. Training School for, notice of, 835.
Ikin, Mr., oxalic acid poisoning, 356.
Indigestion, arsenic in, 352.
Infancy, Combe on the management, of, review of, 33.
Infant feeding and its influence on life, 769.
 mortality, prevention of, 769.
Inman, T., on sour-smelling perspiration in acute rheumatism, 891.
Insane Asylum at Hopkinsville, Ky., burning of, 184.
Insanity, a frightful cause in young men, 636.
 confounding of persons as a symptom of, 686.
 in France, statistics of, 695, 927.
 in Ireland, 934.
Intermittent fever, olive leaves in, 156.
Intermittents, chuguiragua in, 710.
 cinchonine in, 1117.
 nitric acid in, 541.
International exhibition, committee on surgical instruments for the, 957.
Invagination of ileum into colon, 172.
Inversio uteri, reduction of, 1115.
Iodine in acute phlebitis, 544.
 in tubercular meningitis, 1117.
Ireland, statistics of insanity in, 934.
Irish Medical Association, meeting of, 958.
Iron and quinine, a new salt, 712.
 perchloride of, in in-growing toenail, 1092.
Itzigsohn, H., aceton in affections of the larynx, 157.
Ives, Dr., death of, 1137.

JACKSON, DR., case of uterine hæmatocele, 900.
 James, Washington's last illness, 327.
 another letter to a young physician, 993.
 R. M. S., the mountain, review of, 56.
Jago, J., obscure trunk pains, 739.
James, H., premature labor, 698.
Jardin des Plantes, origin of menagerie of, 762.
Jeaffreson, J. C., book about doctors, review of, 577.
Jeannel, Dr, on the taste of cod-liver oil, 350.
Jefferson Medical College, resolutions on Dr. Meigs's resignation, 568.
Jenner, W., appointment of, 571.
Jobert, M., removal of a ball from the cranium, 679.
Jones, Bence, on sugar in urine, 545.
 G. M., digitalis in delirium tremens, 157.
 Mr., death of, 1135.
Journals, new medical, 184, 377, 759.
Jugular vein, internal obliteration of, 550.
Just, O., Jr., statistics of operations on the tongue, 319.

KEATING, W. V., appointment of, as professor, 568.
Kekune oil, 542.
Keller, Dr., empyema and pneumothorax, 363.
Kidd, G. H., pathology of asthma, 1106.
Kidneys, diseases of, accompanied by albuminuria, 887.
Kinesipathy, theory, etc., of, 830.
Klob, J., pathological anatomy of the pancreas, 170.
Knee-joint, dislocation of, intra-uterine, 116.
 ulceration of cartilages of, 175.
Kölliker, A., manual of human microscopic anatomy, review of, 831.
Kousso in tape-worm, 350.
Krassing, Dr., eclampsia as connected with Bright's disease, 133.
Kukui oil, 542.
Kussmaul, A., on epileptiform convulsions, 520.

LABOR, premature, induction of, 608, 1112.
Labors, tedious, quinia in, 523.

Lallemand, M., alcohol and anæsthetics, 707.
Langenbeck, Prof. B., new osteoplastic operation on the nose, 118.
osteoplastic resection of the upper jaw, 1087.
Langlebert, E., distilled water of copaiva, 1119.
La Roche, R., address before Philadelphia Medical Society, 182.
Laryngoscope, Czermak on, 262.
Larynx, aceton in affections of, 157.
Latour, R., collodion in phlegmasia dolens, 897.
Laurence, J. Z., case of sudden death, 163.
Lawes on animal food, 538.
Lawson, Dr., death of, 766.
G., paralysis of ciliary muscle, 688.
L. M., on phthisis pulmonalis, review of, 805.
Laycock, T., morbid pigmentary changes in the skin, 513.
Lead amaurosis, 313.
poisoning, effects of, on the fœtus, 162.
Leared, A., on globularia alypum, 349.
Lebel, M., ergot in blenorrhagia, 352.
Lee, C. C., cancer of the liver, 365.
H., effects of reflex irritation from syphilitic disease of the bones of the skull, 886.
trephining in syphilitic disease of the skull, 320.
Lefort, M., resection of the hip-joint, 682.
Legoyt, M., statistics of insanity, 695.
Leidy, J., human anatomy, review of, 52.
Lente, F. D., ether and chloroform, 679.
Leprosy of the West Indies, 128.
Leucocythæmia, case of, 126.
Leucorrhœa, treatment of, 528.
Lewis, J., quinia in tedious labors, 523.
Liebig, Prof., appointment of, 164.
Lightning, effects of, on man, 1101.
Lingism, theory, etc. of, 830.
Lip, malformation of, four cases in one family, 315.
Lithotomy, écraseur in, 764.
in children, 1091.
in Persia, 318.
recto-vesical, Bozeman's suture in, 121.
Live birth, what constitutes, 1123.
Liver, presence of sugar in, 147.
rare case of disease of, 554.
rupture of common duct of, 723.
sugar-forming function of, 539.
Liverpool fever at Alexandria, 886.
Lives of eminent American physicians, etc., edited by Dr. Gross, notice of, 66.
Locomotory ataxy, 694.
Lumbo-sacral neuralgia simulating uterine affections, 526.
Lumpe, Dr., fibrous tumor of uterus, 340.
Lung, affections of, from bronchitis, 834.
rupture of, 551.
Luxations of shoulder-joint, new mode of reducing, 881.
Lymphatics, new formation of, in puerperal fever, 719.
Lynch, F. J., on ulceration of cartilages of joints, 175.
Lyons, R. D., handbook of hospital practice, notice of, 640.
treatise on fevers, review of, 600.

MACKENZIE, DR., creosote vaginal injections, 135.
Madge, H., uterine hæmatocele, 899.
Madras Medical Journal, 759.
Maisch, J. M., on anacahuite wood, 539.
Maisonneuve, M., reproduction of bones, 684.
Malformation of the heart, with stricture of the pulmonary artery, 922.
Malgaigne's hooks in fractures, 683.
Malmsten, Hr., softening of the medulla spinalis, 167.
Mandl, L., on pulmonary osmose, 347.
Mantegazza, P., zoosperms of the frog, 342.
Marjolin, M., rupture of the lung, 551.
Marotte, Dr., lumbo-sacral neuralgia, 526.
Marsh, Sir H., death of, 382.
Martin, H. A., placenta prævia, 697.
Martinet, M., on disinfectants, 714.
Martyn, Dr., case of diseased liver, 554.
placenta prævia, 698.
Matchett, W. H., injury of the head, with loss of brain, 507.
Matico combined with copaiva, 1118.
Maxson, E. R., treatise on the practice of medicine, review of, 823.
Mayer, Prof., on corpora amylacea, 727.
McLeod, Dr., appointment of, 185.
Mediastinum, empyema, etc. in, 169.
Medical life, wear and tear of, 728.
schools abroad, 569.
and students, 185.
of Canada, 569.
society of Pennsylvania, 571.
students and graduates of 1860–61, 569.
Medulla spinalis, case of softening of, 167.

Meigs, Prof., resignation of, 567.
J. A., on the mensuration of the human skull, 837.
J. F., areolar cancer of the peritoneum, etc., 105.
Meissner, E. A., conceptions in anæmia, 702.
Mémère on deafness, 532.
Memoranda medica, by Hartshorne, notice of, 62.
Mendenhall, G., persulphate of iron in uterine hemorrhage, 132.
Meningitis, tubercular, iodine in, 1117.
Menorrhagia, treatment of, 136.
Menstruation, cutaneous, 515.
Mensuration of the human skull, 837.
Michalowski, Dr., rhus toxicodendron in paralysis, 154.
Michigan University, lectures of, 759.
Microscopic anatomy, Kölliker's, 831.
Midwifery, Churchill on the theory and practice of, review of, 46.
Military surgery, manual of, 642, 978.
maxims of, 425.
works on, 425, 642, 818, 978.
Milk, saccharine fermentation in, 1116.
suppression of, glycerin, etc. in, 337.
Miller, case of ovariotomy, 112.
Milroy, G., quarantine and yellow fever, 690.
Mitchell, S. W., abstracts by, 143, 342, 531, 707, 906.
hepatic abscess opening into the lung, 366.
on rattlesnake bites, 269.
on the rattlesnake venom, review of, 628.
Moodelly, P. S. M., case of ovariotomy, 898
Morgan, J. E., consumption in the Hebrides, 125.
Morel, C., compendium of human histology, review of, 829.
Morland, W. W., work on the urine noticed, 1036.
Morris, G. S., treatment of menorrhagia, 136.
Mortality, infant, prevention of, 769.
Morton, Dr., ether patent, 183.
Mortuary statistics of England, 1100.
Motley, J. T., not a surgeon, 760.
Motorpathy, theory, etc., of, 830.
Mountain, by Jackson, review of, 56.
Movement-cure, theory and practice of, 830.
Mühlig, Dr., penetrating wound of the heart, 940.
Murray, J. J., malformation of the lip in a family, 315.
Muscular contractions from irritation in living man, 152.
poisons, 149.
Museum of comparative zoology, 184.

NATES, sero-sanguineous cyst of, 559.
Navy, Board of Examiners of, 378.
Necrological notices, viz:—
Dr. J. Cobb, 186; Dr. C. Harris, 190; Dr. H. R. Robards, 191; Dr. D. D. Owen, 191; M. Desprès, 191; Dr. C. Coote, 191; Dr. J. W. Francis, 380; Sir H. Marsh, 382; Dr. E. Rigby, 383; Dr. Andrews, 383; Dr. T. Harris, 573; Dr. W. Harris, 573; Dr. F. Tiedemann, 573; Dr. Textor, 574; Dr. E. J. Fountain, 575; Dr. Aran, 575; Dr. F. Adams, 575; Dr. W. Baly, 575; Dr. Lawson, 766; Dr. D. M. Reese, 766; Dr. W. H. Porter, 767; Dr. C. Parry, 959; Mr. H. Gray, 959; Von Ammon, 959; Dr. R. Froriep, 959; Mr. Jones, 1135; Mr. Cusack, 1137; Dr. Ives, 1137.
Negroes, stone in, 185.
Nerves, hypertrophy of connective tissue of, 726.
Nervieux, M., anomaly of the heart, 719.
Nervous system, Brown-Séquard on, notice of, 118.
Neuralgia after parturition, 1108.
Neurotomy, five cases of, 121.
Newhouse, R. B., treatment of menorrhagia, 136.
New York Hospital, pathological cabinet of, 265.
Nitric acid in intermittents, 541.
Nivet, V., the placenta, 525.
Nonat, M., chlorosis, 530.
Nott, J. C., on stone in negroes, 185.
Noyes, J. F., case of recto-vesical lithotomy, 121.
cystic tumors of the vagina, 1114.
Nunn, T. W., inflammation of the breast, 896.
Nuttall, H., neuralgia after parturition, 1108.

OBSCURE trunk pains, 739.
Obstetric statistics, 130.
Obstetrical Society's transactions, notice of, 1037.
Obstetrics, Bedford's work on reviewed, 1035.
Ogilvie, J. F., the Liverpool fever at Alexandria, 886.
Ogle, J., cases of aneurism of aorta, 552, 553.
J. W., a case of recurrent anæsthesia, 517.

Ogle, J. W., softening of the pons Varolii, 360.
Ogston, F., on death by cold, 173.
Ohio State Medical Society Transactions, notice of, 644.
Olive-leaves, extract of, in intermittents, 156.
Onion-juice in ovarian dropsy, 351.
Opium in puerperal peritonitis, 897.
Orbit, pulsating tumors in, 1084.
Orfila, L., quantities in toxicology, 548.
Ormerod, E. L., rupture of the uterus during labor, 132.
Orr, R. S., on lead amaurosis, 313.
Ossification of muscles, 724.
Osteoplastic operation upon the nose, 118.
resection of the jaw, 1087.
Os uteri, cauliflower excrescence of, removed, 527.
in hip-joint disease, 703.
rigidity of, 522.
Otterson, W. C., local remedies in gleet, 666.
Ovarian disease with pregnancy, 700.
dropsy, onion-juice in, 351.
with rupture of the cyst, 898.
Ovaries, diseases of, 417.
Ovariotomy, cases of, 112, 369, 898
in London hospitals, 316.
with recovery, 369.
Ovary, enlarged, prolapsed into the recto-vaginal cul de-sac, 899.
Ovulation during pregnancy, 136.
Owen, D. D., death of, 191.
Oxalic acid, poisoning by, 356.

Packard, J. H., abstracts by, 166, 360, 550, 673, 717, 872, 919, 1084.
case of fracture of patella, 683.
Pallen, M. M , occlusion of uterus, 701.
Pancreas, pathological anatomy of, 170.
Paper hangings, poisoning by, 357.
Paralysis, hypochondriac delirium preceding, 517.
rhus toxicodendron in, 154.
Paré, Ambrose, life of, by Gross, 1059.
Paris hospitals, appointments in, 378.
six months in, 1134.
summer lectures in, 763.
Parturition, neuralgia after, 1108.
Pathological phenomena generalized, notice of, 836.
society (Philadelphia) proceedings, 379.
Paul, M., on lead poisoning, 162.
Pavy, F. W., influence of alkalies on circulation, etc., 908.
on liver-sugar, 539.
Peachy, St. G., spontaneous cure of cataract, 317.
Peckott, M. Th., anchieta salutaris, 710.
Pemberton, O., aneurismal varix following pressure for the cure of aneurism, 881.
Pennsylvania Medical College, resignation of faculty of, 956.
Hospital, pathological museum of, 184.
Peritoneum, areolar cancer of, with hæmatocele, etc., by Dr. J. F. Meigs, 105.
Perrin, M., alcohol and anæsthetics, 707.
Perspiration, sour-smelling, as a symptom of acute rheumatism, 891.
Pessaries, use of, 338.
Pessary, a hurtful agent, 337.
Pfaff, M., on digitalis in cardiac affections, 909.
Pharmaceutical Association, proceedings of, 268.
Phenic acid, in tinea and scabies, 1117.
Philadelphia College of Pharmacy, 570.
Philothalos, wife's domain, 639.
Phlebitis, tincture of iodine in, 544.
uterine, 705.
Phlegmasia dolens, collodion in, 897.
pathological lesion of, 339.
Phthisis, chlorate of potassa in, 351.
review of Lawson on, 805.
Physician's hand-book of practice for 1861, by Elmer, notice of, 64.
visiting list, diary, etc., notice of, 64.
Physiology, human, Dalton's, 449.
Pigmentary changes in the complexion, 513.
Pine-leaf cure in Germany, 572.
Pirrie, W., on favus, 328.
principles, etc. of surgery, notice of, 450.
Placenta, functions of the, 525.
in twin pregnancies, 131.
new sign of detachment of, 335.
removal of, by uterine action alone, 895.
Placenta prævia, 333, 523, 697, 1110, 1111, 1112.
treated by the caoutchouc water pessary, 523.
Plazer, Dr. Von, icterus, etc., 556.
Pneumonia, analysis of one hundred and thirty-three cases of, 322.
Pneumothorax after empyema, case of, 363.
Pockels, Dr., treatment of leucorrhœa, 528.
Pocket dose book, Wythes's, 826.
Poisoning, duty of physicians in, 1123.
Pollak, Dr. lithotomy in Persia, 318.
Pons Varolii, cases of disease of, 889.

Pons Varolii, softening of, three cases, 360.
Porter, Dr. W. H., death of, 767.
Potassa, chlorate of, as a disinfectant, 714.
Potassium, bromide of, properties of, 714.
cyanide of, as a poison, 717.
Pouillet, M., on sulphurous powder, 713.
Power, J. H., anatomy of the arteries, etc., notice of, 448.
appointment of, 957.
Practice of medicine, Maxson's, 823.
Premature labor, induction of, 131.
Prestat, M., wound of the spinal cord, 314.
Prichard, A., case of popliteal aneurism, 683.
Priestley, Dr., treatment of peculiar cases of abortion, 894.
Prisoners, mental condition of, statistics of, 1092.
Prize, subjects for, in Germany, 762.
Prizes of colleges in London, 761.
Professional tricksters, 735.
Prolapsus ani, strychnia in, 707.
of the vagina, 705.
Publications, medical, latest, 1133.
Puerperal epidemic at Wurzburg, 134.
fever, creosote injections in, 135.
with diphtheria, 700.
peritonitis treated with opium, 897.
vaginitis, 705.
Pulmonary osmose, researches on, 347.
Pyæmia, its nature, etc., 676.
Pyocyanine, 765.

QUACKERY in Germany, 572.
Quarantine and epidemics, 690.
Quinia in tedious labors, 523.

RADFORD, T., placenta prævia, 1112.
Radius, luxations of upper end of, 1088.
Rational medicine by Ward, review of, 235.
Rattlesnake bites, treatment of, 269.
venom of, with anatomy, etc. of the organs concerned, 628.
Ray, R., Jr., catalogue of pathological cabinet, etc., notice of, 265.
Raymond, H. H., case of rheumatic fever, 130.
Read, W., placenta prævia, 1110.
Reese, Dr. D. M., death of, 766.
Reil, Dr., on fish poisons, 165.
Resection, reproduction of bones after, 684.
Rheumatic fever, with endocarditis, case of, 130.
Rheumatism, phosphate of ammonia in, 711.
Rheumatism, acute, sour-smelling perspiration as a symptom of, 891.
statistical account of 476 cases of, 893.
Rhus toxicodendron in paralysis, 154.
Ribes, F., work on therapeutical hygiene, review of, 961.
Richardson, Dr., patency of the foramen ovale, 329.
peroxide of hydrogen, 143.
views of on coagulation combated, 538.
Richardson, T. G., prolapsus of the vagina, 705.
Ricord, M., retirement of, 183.
Rienderhoff, Dr., on santonin, 160.
Rigby, E., death of, 383.
Ringer, S., on endocardial murmurs, 686.
Ring-worm, clinical report on, 328.
Robards, H. R., death of, 191.
Robert, M., on fissure of the anus, 508.
Roberts, D. L., placenta prævia, 697.
Robinson, T., congenital deficiency of the diaphragm, 364.
Rodrigues, Dr., cotyledon umbilicus in epilepsy, 154.
Rokitansky, Prof., abortions and uterine polypi, 341.
on movable spleen, 171.
sarcomata of uterus, 557.
Rose, E., the physiological action of santonine and oil of artemisia contra, 910.
Rosenthal, J., influence of the pneumogastric, etc. on the diaphragm, 908.
Roser, Prof., on pyæmia, 676.
Roseville, M. A. De, olive leaves in intermittents, 156.
Routh, C. H. F., infant feeding, etc., review of, 769.

SANDS, DR., fibro-nucleated tumor of the brain, 920.
Sanitary commission, Philadelphia members of, 957.
measures in cities, reports on, 832.
Sansom, Dr., aneurism of the aorta, 169.
Santonine, physiological action of, 910.
properties, etc. of, 160.
Savin, local action of, 911.
Savoyard universities suppressed, 184.
Scabies, phenic acid in, 1117.
Scalp, skull, etc., injuries, etc. of, new work by Prof. Gross, 185.
Scanzoni, Prof., ovulation during pregnancy, 136.
urticaria of female sexual organs, 141.
Schmidt on certain influences of arsenious acid, 537.

Schoeffer, M., on albuminoid substances, 162.
Schottin, E., on creatin, etc., 345.
Schuchardt, Dr., on the effects of aniline, 910.
Schirrous eyeball extirpated, 509.
Scotland, births in, 572.
Sebaceous tumors simulating piles, 316.
Sebastien, Dr., uterine phlebitis, 705.
Sedgwick, W., on sexual limitation in hereditary disease, 1095.
Sédillot, M., on the regeneration of bone, 1087.
Seiler, J., on galvanization by influence, review of, 249.
Sewerage, water supply, etc., report on, 832.
Sexual limitation in hereditary disease, 1095.
Shakspeare, psychology of, 1126.
Shearer, G., a case of leucocythæmia, 126.
Shelton, C. S., treatment of hooping-cough, 905.
Shoulder-joint, new mode of reducing luxations of, 881.
Sibbald, Dr., deposits of nitrate of silver, 727.
Sieveking, E. H., 52 cases of epilepsy analyzed, 514.
Sigmoid flexure, disease of, 1097.
Silver, nitrate of, deposits of, 727.
Simpson, A. R., congenital absence of the gall-bladder, 925.
  intra-uterine small-pox, 342.
  J. Y., sub-involution of the uterus, 336.
  treatment of amenorrhœa, 901.
Sinclair, E. B., use of the forceps, 1107.
Skey, F. C., abdominal or pelvic abscess, 878.
Skin diseases, anchieta salutaris in, 710.
Skinner, W., ossification of muscles, 724.
  T., ferri carbonas effervescens, 1120.
Skull, human, mensuration of, 837.
Slade, D. D., diphtheria, its nature, etc., 330.
  "to what affections of the lungs does bronchitis give origin?" review of, 834.
Small-pox in mother and child, 705, 706.
  intra-uterine, 342.
  in Prussia in 1859, 1101.
Smart, Dr., diseases of Hong Kong, 1104.
  T. T., obstetric statistics, 130.
Smith, H. H., appointment of, 759.
  reduction of luxations of the humerus, 510.
  J. L., infantile erysipelas, 904.
  N. R., new mode of reducing luxations of the shoulder-joint, 881.
Smith, N. R., suspensory apparatus for fractures of the lower extremity, 882.
  S., simulated hip-joint disease, 703.
  W. T., Hunter's doctrines on retroversion reviewed, 338.
Snell, Dr., a symptom of insanity, 686.
Solanum and solanine, 709.
  dulcamara as a poison, 717.
Soldier's friend, 758.
Soyer's culinary campaign, reviewed, 1023.
Spaeth, Dr., placenta in twin pregnancies, 131.
Spinal cord, case of wound of, 314.
Spleen, movable, cases of, 171.
Staples, G. M., veratrum viride in hysteria, 903.
Statistics in the observation of disease, value and fallacy of, 892.
St. Bartholomew's Hospital, income of, 763.
Steadine a substitute for lard, 543.
Stewart, P., the use of pessaries, 338.
St. Mary's Hospital Medical School, announcement of, 957.
Stomach, case of perforation of, 689.
Stone, clinical experience in the treatment of, 506.
  in negroes, 185.
  urethrotomy for, 511.
Stricker, W., effects of lightning on man, 1101.
Stricture of the urethra, by Dupierris, notice of, 63.
  by Wade, notice of, 1030.
Stromeyer, L., military surgery, review of, 425, 978.
Stürzwage on arsenious acid, 537.
Sudden death without adequate post-mortem appearances, 163.
Sugar in the liver, presence of, 147.
Sulphurous powder, 713.
Summary of medical science, Wells's, notice of, 643.
Sun-stroke at Lucknow, 124.
Surgeon-General, appointment of, 759.
Surgeons, Royal College of, prize of, 572.
Surgery, Gill's epitome of, review of, 60.
  military, works on, 642, 818, 978.
  Pirrie's work on, 450.
Surgical cases of Prof. Gross, 656.
Suspensory apparatus for fractures of the lower extremity, 882.
Sweeting, R., leprosy of the West Indies, 128.
Sydenham Society, (New,) notice of, 761.
  publications of, 378, 1134.
Syme, Mr., cases of gluteal aneurism, 879.

Syme, Mr., honor conferred on, 1135
Syphilitic disease of the liver, 722.
 of skull-bones, effects of reflex nervous irritation produced by, 886.

TAPE-WORM, kousso in, 350.
Taylor, A. S., on the detection of arsenic, 913.
 medical jurisprudence, notice of, 1037.
 C. F., theory and practice of the movement-cure, review of, 830.
Tendons, reparative process in, 444.
Tenner, A., on epileptiform convulsions, 520.
Tetanus, traumatic, case of recovery from, 675.
Textor, Dr., death of, 574.
Thapsia plaster, 545.
Thermo-therapeia, 353.
Thibierge, Dr., vegetations on the genitals, 704.
Thiercelin, M., woorara in epilepsy, 352.
Thomas, T. G., on placenta prævia, 333.
Thompson, E. S., invagination of ileum into colon, 172.
Thomson, J. B., statistics of the mental condition of prisoners, 1092.
Thorn, W., induction of premature labor, 131.
Tiedemann, F., death of, 573.
Tilt, E. J., treatment of uterine inflammation, 528.
Tinea, phenic acid in, 1117.
 tonsurans, clinical report on, 328.
Title, curious, 760.
Toe-nail, ingrowing, perchloride of iron in, 1092.
Tongue, statistics of operations on, 319.
Toxicology, question of quantities in, 546.
Toynbee, J., on caries of petrous bone, 685.
Transposition of all the thoracic and abdominal viscera, 926.
Trephining in syphilitic disease of the skull, 320.
Trifolium in fœno, a remedy for hooping-cough, 906.
Tripler, Dr., notice of, 760.
 hand-book for the military surgeon, review of, 818.
Trousseau, M., emboli of pulmonary artery, 721.
Trunk pains, obscure, 739.
Tumor, fibro-nucleated, of the brain, 920.
Turkey from a medical point of view, 486.
Turkish bath in insane asylums, 764.
Turner, W. M., the pessary injurious, 337.
 vomiting in pregnancy, 699.
Turpentine as an anæsthetic, 572
Typhoid fever, modes of death, 1094.
Typhus and typhoid fevers, identity of, 128.
 in Liverpool, 690.
 reflections on, 861

UMBILICAL CORD, shortness of, 142.
 funis, prolapsed, instrument for reduction of, 895.
Upas, a supposed new species of, 537.
Uræmic intoxication, 536.
Urethra, irritation of, in females, 903.
 stricture of 63, 1030.
Urethrotomy for stone in the bladder, 511.
Urinary calculus in a child, 669.
Urine, Morland's work on noticed, 1036.
 sugar in, 545.
Urticaria of female sexual organs, 141.
Ustilago madis, a cause of abortion, 1121.
Uterine glands in sarcomata of uterus, 557.
 hæmatocele, case of, 899, 900.
 inflammation, injections, etc., in, 528.
Utero-gestation lasting thirty-two years, 699.
Uterus, cancer of, 136.
 fibrous tumor of, 340.
 fungosities of, 900.
 fungus hæmatodes of, 369.
 inversion of, 1115.
 occlusion of, 701.
 polypi of, contributions to history of, 341.
 retroversion, etc. of, Hunter's views reviewed, 338.
 rupture of, 132, 523.
 with enlarged and prolapsed ovary, 899.
 sub-involution of, after delivery, 336.

VAGINA, cystic tumors of, 1114, 1115.
 occlusion of, 701.
Valedictory, editorial, 1125.
Valerian, essence of, 711.
Vedder, A. M., perforation of appendix vermiformis, 172.
Vee, M., new way of giving chloroform, ether, etc., 1020.
Vegetations on the genitals, 704.
Vella, M., woorara and strychnine, 358.
Vénot, M., onion-juice in ovarian dropsy, 351.
Venous circulation, causes of, 453.
Veratrum viride in hysteria, 903.

Vesalius, life and writings of, review of, 1.
Virchow, R., cellular pathology, review of, 385.
Vivenot, R. Von. Jr., influence of change of atmospheric pressure, 907.
Vocal cords, warts on, 717.
Volunteer service, medical staff of, 759.
Vomiting in pregnancy, 699.

WADE, R., stricture of the urethra, reviewed, 1030.
Wagner, M. E., lymphatics in puerperal fever, 719.
R., on the function of the brain 534.
Wahu, M., on ingrowing toe-nail, 1092.
Walker, T. O., Jr., disease of the sigmoid flexure, 1097.
Waller, A., experiments on cutaneous absorption, 708.
War, impending, 757.
Ward, S. H., rational medicine of, review of, 235.
Waters, A.T.H., researches on asphyxia, the hot bath, etc., 884.
Washington, last illness of, 327.
Wear and tear of medical life, 728.
Weber, H., cases of disease of the pons Varolii, 889.
Wells, W. S., summary of medical science, notice of, 643.
West, R. U., action of ergot on the fœtus, 1109.
White, J. H., thirty-two years' gestation, 699.
Wife's domain, notice of, 639.
Wilks, Dr., obscure cases of cancer of the œsophagus, 920.
Wilson, E., Thermo-therapeia, 353.
J. G., instrument for reducing prolapsed umbilical funis, 895.
Winslow, F., Medical Critic, notice of, 452.
Wise, T. A., on Hindu medicine, review of, 616.
Women, hospital for diseases of, 184.
Hodge on diseases of, review of, 220.
practitioners in ancient times, 372.
of midwifery in modern times, 560.
Wood, John, stone extracted by urethrotomy, 511.
Woodman, Mr., cancer of the œsophagus, 365.
Woodward, J. J., serous cysts of the breast, 923.
Woorara in epilepsy, 352.
antagonistic to strychnine, 358.
Wounded at Bull Run, visit to the, 949.
Wythes, J. H., physician's pocket dose book, review of, 826.

YEAR-BOOK of American medical literature, 571.
Yellow fever in Jamaica, 690.
in ships of war, 690.
Youmans, J., intra-uterine dislocation of the knee-joint, 116.
Young, E. P., transposition of all the thoracic and abdominal viscera, 926.

ZIEGLER, DR., soldier's friend, 758.
Zimmerman on Richardson's hypothesis, 538.
Zoospermes of the frog, 342.
Zymotic disease, spread of, 690.

